# Syphilitic Synovitis in Children

**Published:** 1904-11-19

**Authors:** 


### SYPHILITIC SYNOVITIS IN CHILDREN.

In a paper on this subject read to the Medico-Chirurgical Society of Edinburgh, Dr. Melville Dunlop stated that this condition is deserving of wider recognition, as it is probably much more common than is usually supposed; many cases regarded as tubercular arthritis or sub-acute rheumatism being really due to syphilis. Two forms are met with:—(1) Synovitis following epiphysitis; (2) True chronic syphilitic synovitis. The first variety is associated with congenital syphilis, and occurs in infants during the first three months of life. It is more frequent in the upper than the lower limbs, chiefly affecting the elbows, wrists, and knees. The affection depends on a gummatous condition of the epiphysis, and separation at the epiphyseal line may occur, with considerable effusion into the joint, with pain and disuse of the limb. There is rarely true crepitus, and union is usually secured, while suppuration is very rare. Under mercurial inunction rapid improvement takes place.

The second variety is both chronic and insidious. It represents a late tertiary development and occurs between the ages of eight and 15 years, though it has been seen as late as 18 years of age. The symptoms are those of an effusion into the joint accompanied by little or no pain or bony enlargement. The synovial membrane is rarely much thickened, destructive changes are absent, and the condition is not followed by muscular atrophy. The lesions tend to be multiple, the knees and elbows being chiefly affected. There is a marked tendency to a symmetrical distribution, though it is usual for one joint to be involved some weeks or even months before its fellow. Of 16 cases observed by Dr. Dunlop both knees were involved in 12, while no affection of the hip-joint was noted. This form of synovitis is associated with other of the later manifestations of syphilis, more especially with interstitial keratitis, which, like the joint lesions, tends to be bilateral. Keratitis was present in 12 of the 16 cases noted above.

The joint condition is very chronic and relapses are frequent. The synovial membrane is usually the site of gummatous changes, either coarse or microscopic. The joints do not tend to suppurate, and it is probable that cases of supposed tubercular arthritis ending in spontaneous recovery have been cases of this nature. In these cases, treatment with iodide of potassium and mercury has proved more efficacious than the use of mercury alone, and may be assisted by the inunction of mercurial ointment over the affected joints.

